# HLA-B^∗^57 and Gender Influence the Occurrence of Tuberculosis in HIV Infected People of South India

**DOI:** 10.1155/2011/549023

**Published:** 2011-08-25

**Authors:** Latha Jagannathan, Mrinalini Chaturvedi, Bhuthaiah Satish, Kadappa Shivappa Satish, Anita Desai, D. K. Subbakrishna, Parthasarathy Satishchandra, Ramasamy Pitchappan, Kamala Balakrishnan, Paturu Kondaiah, Vasanthapuram Ravi

**Affiliations:** ^1^Rotary-TTK Blood Bank, Bangalore Medical Services Trust (BMST), Bangalore 560075, India; ^2^HIV Services, Seva Free Clinic, Bangalore 560042, India; ^3^Department of Neurovirology, National Institute of Mental Health and Neuro Sciences (NIMHANS), Bangalore 560029, India; ^4^Department of Biostatistics, National Institute of Mental Health and Neuro Sciences (NIMHANS), Bangalore 560029, India; ^5^Department of Neurology, National Institute of Mental Health and Neuro Sciences (NIMHANS), Bangalore 560029, India; ^6^Department of Immunology, School of Biological Sciences, Madurai Kamaraj University, Madurai 625021, India; ^7^HLA Laboratory, College of Medicine, University of Cincinnati, Ohio 45267-0550, USA; ^8^Department of Molecular Reproduction, Development and Genetics (MRDG), Indian Institute of Science (IISc), Bangalore 560012, India

## Abstract

*Background*. Substantial evidence exists for HLA and other host genetic factors being determinants of susceptibility or resistance to infectious diseases. However, very little information is available on the role of host genetic factors in HIV-TB coinfection. Hence, a longitudinal study was undertaken to investigate HLA associations in a cohort of HIV seropositive individuals with and without TB in Bangalore, South India. *Methods*. A cohort of 238 HIV seropositive subjects were typed for HLA-A, B, and DR by PCR-SSP and followed up for 5 years or till manifestation of Tuberculosis. HLA data of 682 HIV Negative healthy renal donors was used as control. *Results*. The ratio of males and females in HIV cohort was comparable (50.4% and 49.6%). But the incidence of TB was markedly lower in females (12.6%,) than males (25.6%). Further, HLA-B*57 frequency in HIV cohort was significantly higher among females without TB (21.6%, 19/88) than males (1.7%, 1/59); *P* = 0.0046; OR = 38. CD4 counts also were higher among females in this cohort. *Conclusion*. This study suggests that HIV positive women with HLA-B*57 have less occurrence of TB as compared to males.

## 1. Introduction

India has the world's highest number of Human immunodeficiency virus (HIV) infections for any country outside Africa with estimated 2.47 million infections. The commonest mode of transmission of infection is through heterosexual contact and the majority of the HIV infections (89%) are in the age group of 15–49 years. Karnataka is one of the four states in Southern India which has a high prevalence of HIV infection (0.81%), especially amongst ante-natal mothers who are considered as low-risk group [1]. A recent study from South India has indicated that HIV 1 clade C accounts for over 90% of all HIV infections in the country [2]. Amongst the 256 samples tested from South India, 253 were identified as HIV 1 clade C using a subtype-specific PCR [2]. The predominant opportunistic infection noted among AIDS patients in India is tuberculosis (TB) and it is the most potent risk factor associated with disease progression. An HIV positive person is six times (50 to 60% lifetime risk) more likely to develop TB disease as compared to an HIV negative person (10% lifetime risk) [3]. Notwithstanding such a high risk of developing TB, it has been observed by us that there is a subset of HIV infected individuals who do not develop TB over a number of years (unpublished observations at Seva free clinic, an HIV care centre at Bangalore). Taken together, this suggests that certain “innate factors” could indeed influence the manifestation of TB in HIV infected individuals. Interactions between HIV and the innate immune system have recently caught the attention of several researchers [4]. In particular, the importance of differential Toll-Like Receptor 7 (TLR7) mediated signaling leading to increased interferon-*α* production in plasmacytoid dendritic cells (pDCs) noted in HIV infected women as compared to men thereby explaining how viral loads are better controlled in women [5].

There is substantial epidemiological evidence that host genetic factors such as Human Leukocyte Antigens (HLAs) and closely linked genes of the Major Histocompatibility Complex (MHC) as well as non-MHC factors such as chemokine receptors are important determinants of susceptibility or resistance to infectious diseases [6–8]. Human Leukocyte Antigens are central to the recognition and presentation of pathogens to the immune system and therefore are a fundamental part of the human immune system. Several studies on HLA and disease association with HIV have been reported in different ethnic populations [6–8]. Little information is available on HIV infected individuals in South India with special reference to the incidence of TB and association with HLA. Therefore, this study was undertaken in a cohort of HIV positive individuals to investigate the association of HLA and TB.

## 2. Materials and Methods

### 2.1. Study Design

The study was prospective in design and included 263 drug naive HIV seropositive individuals who attended Seva Free Clinic (HIV care centre at Bangalore). The study protocol was approved by the Institutional Ethical Committees of Seva clinic and Bangalore Medical Services Trust. Most patients visited the clinic with the onset of symptoms and the duration of HIV infection was thus self-reporting. HIV infection was confirmed by demonstration of anti-HIV antibodies in serum as per the National AIDS Control Organization's guidelines using rapid HIV tests. All subjects underwent a detailed clinical evaluation by the treating physicians (Bhuthaiah Satish, Kadappa Shivappa Satish, and Parthasarathy Satishchandra). TB was diagnosed in the study subjects based on clinical examination and/or routine laboratory investigations such as sputum smear examination, X-ray chest, and haemogram. Sputum smear and/or culture positivity was considered as confirmatory evidence of TB, whilst chest X-ray, raised erythrocyte sedimentation rates and response to anti-TB treatment were considered highly suggestive of TB. After obtaining informed consent, subjects were recruited into the study and referred to the National Institute of Mental Health and Neuro-Sciences (NIMHANS) for CD4 enumeration. Those subjects who did not any confirmatory and/or clinical evidence of TB were followed up for a period of five years or till manifestation of TB, whichever was earlier. During the follow-up period, 25/263 HIV positive individuals were excluded from the study as their CD4 counts declined below 200 cells/*μ*L and therefore had to be initiated on antiretroviral therapy. No separate controls were recruited for this study, However, the sociodemographic and HLA frequency distribution details available in a healthy renal donor database with BMST, Bangalore, were used for comparative analysis and interpretation of the data obtained from study subjects. None of these donors were HIV positive and showed any signs of active systemic tuberculosis upon clinical examination. Their age, sociodemographic features, and ethnic background (Dravidian race) were comparable to those of the HIV infected group.

### 2.2. HLA Typing

HLA A, B, and DR typing was carried out at Bangalore Medical Services Trust Immunophenotyping laborator. HLA typing for the HIV positive cohort was done by PCR-SSP low-to-intermediate resolution typing employing commercial kits (Genovision, Biotest, and One Lambda, USA) according to the manufacturer's instructions. We compared the frequency distribution of HLA antigens among the HIV infected subjects with that of a 682 healthy renal donor database on South Indians available at our center. HLA typing for some of the renal donors was done by serological methods employing commercial kits (One Lambda, USA). The rest of the controls were typed by PCR-SSP methods. Briefly, 2 mL of whole blood was collected in ethylenediamine tetra acetic acid**- (**EDTA-) coated vaccutainer tubes and DNA extracted using a commercial DNA extraction kit (Qiagen, GmbH, Germany). The purified DNA was added to the master mix containing Taq polymerase and dNTP-buffer, dispensed into the 96-well Micro SSP DNA Typing tray, which was precoated with primer pairs specific for the different HLA Class I and II, alleles, and amplified by Polymerase Chain Reaction. The amplified DNA fragments were separated using agarose gel electrophoresis and visualized by staining with ethidium bromide and exposure to UV light. Interpretation of the results was based on presence or absence of a specific amplified DNA fragment. Results were interpreted using specific interpretation work sheet and specific software provided by the kit manufacturer. The HLA typing was carried out for the following alleles: twenty two HLA Class I A locus antigens (1, 2, 3, 10, 11, 23, 24, 25, 26, 29, 30, 31, 32, 33, 34, 36, 43, 66, 68, 69, 74, and 80), thirty six HLA Class I B locus antigens (7, 8, 12, 13, 15, 16, 18, 21, 22, 27, 35, 37, 38, 39, 41, 42, 44, 45, 47, 48, 49, 50, 51, 52, 53, 55, 56, 57, 58, 60, 61, 62, 63, 71, 75, and 81), and eighteen HLA Class II DR locus antigens (1, 3, 4, 7, 8, 9, 10, 11, 12, 13, 14, 15, 16, 17, and 18).

### 2.3. CD4 Counts

 CD4 counts were enumerated at the WHO accredited laboratory at the Department of Neurovirology, NIMHANS, using a nonlysis method on a Fluorescent Activated Cell Sort Count (FACSCount, Becton Dickenson, USA). Briefly, 50 *μ*L each of whole peripheral venous blood was pipetted into a pair of reagent tubes containing the CD4/CD3 and CD8/CD3 fluorescent-labeled monoclonal antibodies, respectively. The sample was vortexed for a few seconds and incubated at room temperature for one hour. Following the incubation period, 50 *μ*L of formalin fixative solution was added to each of the reagent tube pairs and the counts were analyzed on the FACS count machine using the specialized software provided by the manufacturer.

### 2.4. Statistical Analysis

 The statistical analysis was done using SPSS 15.0 and Epistat software. The tests used were Chi-square test, Fisher's exact probability test, and Student's *t*-test, wherever necessary Odds Ratios were computed. The level of significance was fixed at 0.05.

## 3. Results

### 3.1. Socio-Demographic Profile of Study Population

 Amongst the 238 subjects enrolled in this study, 120 (50.4%) were males and 118 (49.6%) were females. Of these, 161 were from lower socioeconomic strata, 46 from middle, and 1 from upper socioeconomic strata. The socioeconomic status data for the rest (30 individuals) was not available. 74 of the 118 females gave their occupation as housewife. The age of the HIV positive subjects ranged from 23 to 72 years with a median age of 30 years. Most patients visited the clinic to seek medical intervention for signs and symptoms of illness. The precise duration of HIV infection could not be determined precisely as they were either referred from other health care facilities or sought medical consultation voluntarily. The duration of HIV infection was therefore estimated as at least from the first date of attendance at Seva clinic. This ranged from 1 to 12 years and in 101/238 it ranged from 4 to 5 years prior to recruitment in this study. 

Amongst the 682 apparently healthy renal donor populations whose socio-demographic details were available in the BMST database, it was observed that the age ranged from 21 to 70 years with a median of 39 years and a male : female ratio of 1.21 : 1. All the renal donors were from South India and from a similar ethnic origin as those study subjects.

### 3.2. Incidence of TB in HIV Positive Subjects

Table 1 depicts the details of occurrence of TB in the study population. Amongst the 238 subjects enrolled into this study 83 (34.9%) were diagnosed to have TB (56 males and 27 females) at the time of recruitment. Amongst the 83 subjects diagnosed to have TB, 13 were positive for acid fast bacilli (AFB) on sputum (*n* = 9) and CSF (*n* = 4) smear examination, 24 had typical findings of pulmonary TB on X-ray examination (which included 4 with pleural effusion), 11 were positive for AFB on fine needle aspiration cytology of lymph nodes, 3 had evidence of abdominal TB, and the remaining 32 showed excellent clinical response top anti-TB treatment (resolution of abnormal X-ray chest findings or lymph node enlargement). In the 155 HIV positive subjects who had no evidence of TB at the time of recruitment, eight developed TB during the five-year follow-up period (5 males and 3 females). On the other hand, the gender distribution amongst the 147 subjects who did not manifest TB at recruitment or during the subsequent follow-up period showed that women constituted 60% (88/147) while males accounted for 40% (59/147). Overall, the incidence of TB was markedly lower among female subjects (25.42%; 30/118) as compared to male subjects (50.83%; 61/120).

### 3.3. Comparison of HLA Frequencies among the HIV Positive Cohort and Healthy Renal Donors

 The following Class I and Class II, HLA Antigens were identified in the samples in our study: HLA A: 1, 2, 3, 10, 11, 23, 24, 26, 29, 30, 31, 32, 33, 34, 36, 68, and 74; HLA B: 7, 8, 12, 13, 15, 18, 21, 27, 35, 37, 38, 39, 41, 42, 44, 47, 48, 49, 50, 51, 52, 53, 55, 56, 57, 58, 60, 61, 62, 63, 71, 75, and 81; HLA DR:1, 3, 4, 7, 8, 9, 10, 11, 12, 13, 14, 15, 16, 17, and 18.

Tables 2(a), 2(b), and 2(c) depict the frequency distribution of the HLA A, B, and DR alleles, respectively, among the healthy renal donors and the HIV positive subjects. Interestingly, HLA-A*2 (31%), HLA-B*35 (26%), and HLA-DRB1*15 (20%) were found to be the most frequently occurring alleles in both HIV positive cohort and the healthy renal donors. Amongst the healthy renal donors, the incidence of individual HLA antigens did not show any significant differences between males and females. With respect to HIV positive cohort the following observations were made: (i) frequency of HLA-A*2 was higher among TB positive women (Table 2(a)), (ii) frequency of HLA-B*35 was lower among TB negative women, (iii) HLA-B*52 and HLA-B*62 alleles were not detected among TB positive women, (iv) frequency of HLA-B*61 was higher among TB positive women (Table 2(b)), (v) HLA-DRB1*4 and HLA-DRB1*15 frequencies were higher among HIV positive subjects as compared to the healthy renal donors, and (vi) frequency of HLA-DRB1*8 was higher among TB positive men (Table 2(c)). However, none of these differences were statistically significant. On the other hand, there was a significantly different distribution of HLA-B*57 among HIV positive TB negative female subjects as compared to HIV positive males as well as male and female healthy renal donors (controls).

### 3.4. Frequency Distribution of HLA-B*57 (Table 2(b))

Among the 682 healthy, HIV negative, renal donors, the frequency distribution of HLA-B*57 in the males and females was comparable (9.48% and 12.77%, resp.). The incidence of HLA-B*57 among HIV Positive males was low, being only 4.2% (*n* = 5), but the difference as compared to the renal donor data was not statistically significant. Though the proportion of HIV positive females with HLA-B*57 was more (17.8%, *n* = 21) as compared to 12.77% among healthy female renal donors, it did not reach statistical significance (Chi-square =1.50; *df* = 1 *P* > 0.05). The frequency of HLA-B*57 amongst HIV positive males with TB (6.55%; 4/61) and females with TB (6.66%; 2/30) showed no statistical difference. However, the frequency distribution of HLA-B*57 in HIV positive, TB Negative females was 21.59%, (19/88), significantly higher (*P* = 0.0046 with an Odds Ratio of 3.80) than that in HIV positive TB negative males (1.7%; 1/59). Thus the frequency distribution of HLA-B*57 antigen among the HIV population was proportionately more amongst women who did not develop TB as compared to men who did not develop TB. In other words, the relative risk (RR) of HIV positive HLA-B*57 females developing TB was significantly lower (0.26) as compared to males (4.07). When calculated with Haldane-modified Woolf's formula the RR of HIV positive HLA-B*57 females developing TB was significantly lower (0.31) as compared to males (3.87).

### 3.5. CD4 Counts

 The CD4 counts were estimated in 227 subjects (115 females and 112 males) and ranged from 2 to 1155 /*μ*L. In 11 HIV positive subjects (3 females and 8 males) CD4 counts were not estimated. Overall females had higher CD4 counts than males with 26 females and 14 males having CD4 counts >500/*μ*L, 66 females and 57 males from 200 to 500/*μ*L, and 23 females and 41males <200/*μ*L. HIV positive individuals with TB had lower CD4 counts than HIV positive individuals without TB.

### 3.6. CD4 Counts in Subjects with HLA-B*57

 Amongst the TB positive HLA-B*57 subjects, CD4 counts in 2 females were <200/*μ*L while in 3 out of 4 males they were from 201 to 500/*μ*L and >501/*μ*L in 1out of 4 males. Amongst the TB negative HLA-B*57 subjects, CD4 counts in 1 out of 19 females were less than 200/*μ*L, from 201 to 500/*μ*L in 7 out of 19 females, and >501 *μ*L in 11/19 females. The lonly HLA-B*57 male who was TB negative had a CD4 count of 374/*μ*L. Overall CD4 counts in the HLA-B*57 females were higher than those in HLA-B*57 males.

## 4. Discussion

Several studies in different ethnic populations have noted associations between HLA alleles and HIV infection disease progression to AIDS. These studies have demonstrated that the host immune response to HIV infection is influenced by the MHC repertoire of the individual. While some HLA alleles notably HLA-B57 and HLA-B27 were associated with favourable outcomes, others including HLA-B35 and HLA-B22 were associated with unfavourable outcomes [6–9]. Of the many alleles implicated, HLA-B57 has presented a remarkably consistent protective effect in HIV positive individuals in various ethnic populations of the world resulting in low viremia, high CD4 counts, and a Long-Term Nonprogressor (LTNP) state [10, 11]. Both innate and adaptive protective immune mechanisms play a part in Long-Term Nonprogressors, mediated by Cytotoxic T Lymphocyte (CTL) and Natural Killer (NK) cell responses in the context of HLA alleles. HLA B*57-restricted HIV-1-specific CTL responses and protective Killer Immunoglobulin-like Receptor (KIR) alleles in combination with the HLA-B*57 alleles have been demonstrated in LTNPs [12–14]. Other related HLA genes have been shown to contribute to the protective effect mediated by sharing of HLA-B57/B58-restricted CTL epitopes [15, 16]. Several studies have noted that there is significant correlation between HIV-1-specific CD8 T-cell proliferation and HIV replicative capacity, resulting in low viral load and therefore slower progression [17–19]. A study in France in an HIV-1 cohort of slow and rapid progressors found among others an association of HLA DR11 with rapid progression but only among women [7]. While some studies have noted a slower disease progression but a higher rate of death in HIV positive women than in men [20], others have noted a faster disease progression to AIDS [21]. 

Several studies have also investigated the possible role of HLA alleles in the incidence and progression of TB. For instance, some studies from India and other countries have reported different HLA alleles that are associated with incidence and progression of TB [6, 22–24], particularly the association of DRB*1501 with advanced disease and failure to respond to drug therapy. Other studies from India and elsewhere have noted a lower incidence of TB disease among adult females than in adult males, compared to children and adolescents. Smoking has been implicated in the increased incidence of TB among men and gender inequalities and socioeconomic factors for the differences in the epidemiology of TB, HIV, and other diseases in men and women [25–29]. Nevertheless biological factors also have been seen to be associated with the gender differences of these diseases. All these studies have contributed to the better understanding of the possible role of HLA genes in HIV disease progression and/or occurrence of TB in the population. However none of them have addressed the important aspect of HIV-TB coinfections. Since TB is one of the common opportunistic infections encountered in HIV infected subjects, studying the possible role of host genetic elements involved in the occurrence of TB is critical. Therefore, in this study we undertook HLA typing in a cohort of HIV infected individuals with and without TB to investigate whether host genetics would explain the paradoxical clinical observation of absence of TB amongst HIV infected subjects in South India. 

In our HIV positive study population, the incidence of TB among females was significantly lower than in males. Although there was a higher incidence of TB among the men who reported tobacco use (23/45 TB positive males and data related to smoking was unavailable in the remaining 16 TB positive males), it was not statistically significant. Therefore, smoking alone cannot explain the higher incidence of TB among the men in our study. The present study on HLA association in an Indian HIV positive cohort confirms the LTNP protective effect of HLA-B57, which has been reported in various parts of the world [9–14]. The proportion of HIV positive females with HLA-B*57 who did not develop TB in our study was significantly high (*P* = .0046). This observation assumes greater significance given that the male-to-female ratio in the HIV positive cohort was comparable (50.4% males to 49.6% females). The females with HLA-B*57 also possessed higher CD4 counts. It is possible that this may be indicative of HLA-B*57 having a protective effect in females with HIV and slower progression of HIV disease. Yet, this protective influence of HLA-B*57 was not clearly apparent in the male HIV positive individuals. Hormonal influence on noninfectious diseases like cardiovascular disorders is known. But some studies on HIV disease progression, including our own, are suggestive of hormone-mediated adaptive immune responses in the control of infectious diseases [9]. Alternatively, the higher CD4 counts in females may be because of the lower rates of TB in HIV positive women noted in this study, especially because the duration of HIV infection is unknown in our subjects.

 Mutations in HIV-1 due to founder effects and HLA class I-mediated immune mutations contribute to viral gene diversity, which in turn may impact the HLA diversity at the population level [30–32]. The HLA A1-B57 haplotype is of greater frequency in the Indian population suggestive of a possible survival advantage [33]. HLA-B*57 is present in a significant proportion of the population in India and in our control sample it was 11.1% (data not presented). The HIV pandemic in India is still in a state of expansion. In conclusion, this initial study highlights the association of gender, presence of HLA-B*57, and slow progression of HIV infection. However, larger prospective studies are needed to clearly define the role played by HLA mediated and other non-HLA immune mechanisms for susceptibility (risk) or protection which can be used for immunogenetic profiling, risk assessment, therapeutic decisions, and for future vaccine development and treatment. 

## Figures and Tables

**Table 1:** Occurrence of TB amongst HIV positive subjects.

HIV positive subjects (*n* = 238)	Males (*n* = 120)	Females (*n* = 118)
No TB either at recruitment or during follow-up	59 (49.16%)	88 (74.57%)
TB positive individuals in the study		
(i) At recruitment	56	27
(ii) During follow-up	5	3

Total	61	30

**Table:** (a)

HLA A	HIV positive females		HIV positive males		Healthy renal donors	
	TB neg. F	TB pos. F	TB neg. M	TB pos. M	Females	Males
	*n* = 88	*n* = 30	*n* = 59	*n* = 61	*n* = 376	*n* = 306
1	29 (33.0%)	9 (30.0%)	14 (23.7%)	15 (24.6%)	84 (22.3%)	65 (21.2%)
2	27 (30.7%)	12 (40.0%)	20 (33.9%)	19 (31.1%)	115 (30.6%)	95 (31.0%)
3	10 (11.4%)	3 (10.0%)	10 (16.9%)	7 (11.5%)	54 (14.4%)	32 (10.5%)
10	1 (1.1%)	0 (0.0%)	0 (0.0%)	0 (0.0%)	0 (0%)	0 (0%)
11	23 (26.1%)	7 (23.3%)	13 (22.0%)	17 (27.9%)	96 (25.5%)	82 (26.8%)
23	1 (1.1%)	0 (0.0%)	0 (0.0%)	1 (1.6%)	2 (0.5%)	10 (3.3%)
24	20 (22.7%)	8 (26.7%)	16 (27.1%)	20 (32.8%)	97 (25.8%)	79 (25.8%)
25	0 (0.0%)	0 (0.0%)	0 (0%)	0 (0%)	1 (0.3%)	1 (0.3%)
26	5 (5.7%)	2 (6.7%)	3 (5.1%)	5 (8.2%)	23 (6.1%)	23 (7.5%)
29	2 (2.3%)	4 (13.3%)	3 (5.1%)	3 (4.9%)	17 (4.5%)	10 (3.3%)
30	1 (1.1%)	0 (0.0%)	1 (1.7%)	2 (3.3%)	10 (2.7%)	14 (4.6%)
31	8 (9.1%)	1 (3.3%)	6 (10.2%)	1 (1.6%)	28 (7.4%)	28 (9.2%)
32	4 (4.5%)	1 (3.3%)	4 (6.8%)	3 (4.9%)	17 (4.5%)	10 (3.3%)
33	15 (17.0%)	5 (16.7%)	11 (18.6%)	11 (18.0%)	93 (24.7%)	72 (23.5%)
34	0 (0.0%)	0 (0.0%)	1 (1.7%)	0 (0.0%)	0 (0%)	1 (0.3%)
36	0 (0.0%)	0 (0.0%)	1 (1.7%)	0 (0.0%)	2 (0.5%)	5 (1.6%)
43	0 (0.0%)	0 (0.0%)	0 (0%)	0 (0%)	1 (0.3%)	0 (0%)
66	0 (0.0%)	0 (0.0%)	0 (0%)	0 (0%)	0 (0%)	2 (0.7%)
68	13 (14.8%)	6 (20.0%)	7 (11.9%)	9 (14.8%)	51 (13.6%)	32 (10.5%)
74	0 (0.0%)	0 (0.0%)	0 (0%)	0 (0%)	2 (0.5%)	5 (1.6%)
80	0 (0.0%)	0 (0.0%)	0 (0%)	0 (0%)	1 (0.3%)	0 (0%)

**Table:** (b)

	HIV positive females		HIV positive males		Healthy renal donors	
HLA B	TB neg. F	TB pos. F	TB neg. M	TB pos. M	Females	Males
	*n* = 88	*n* = 30	*n* = 59	*n* = 61	*n* = 376	*n* = 306

7	18 (20.5%)	5 (16.7%)	13 (22.0%)	10 (16.4%)	71 (18.9%)	48 (15.7%)
8	4 (4.5%)	4 (13.3%)	2 (3.4%)	4 (6.6%)	5 (1.3%)	13 (4.2%)
13	2 (2.3%)	1 (3.3%)	1 (1.7%)	4 (6.6%)	23 (6.1%)	28 (9.2%)
15	2 (2.3%)	1 (3.3%)	2 (3.4%)	0 (0.0%)	7 (1.9%)	13 (4.2%)
18	0 (0.0%)	1 (3.3%)	2 (3.4%)	4 (6.6%)	12 (3.2%)	12 (3.9%)
21	4 (4.5%)	2 (6.7%)	3 (5.1%)	2 (3.3%)	0 (0.0%)	4 (1.3%)
27	5 (5.7%)	1 (3.3%)	2 (3.4%)	1 (1.6%)	9 (2.4%)	12 (3.9%)
35	15 (17.0%)	8 (26.7%)	18 (30.5%)	20 (32.8%)	104 (27.7%)	75 (24.5%)
37	9 (10.2%)	3 (10.0%)	4 (6.8%)	8 (13.1%)	26 (6.9%)	26 (8.5%)
38	0 (0.0%)	0 (0.0%)	2 (3.4%)	3 (4.9%)	10 (2.7%)	8 (2.6%)
39	0 (0.0%)	1 (3.3%)	0 (0.0)%	1 (1.6%)	3 (0.8%)	8 (2.6%)
41	0 (0.0%)	0 (0.0%)	0 (0.0)%	1 (1.6%)	1 (0.3%)	4 (1.3%)
42	1 (1.1%)	0 (0.0%)	0 (0.0)%	0 (0.0%)	1 (0.3%)	3 (1.0%)
44	7 (8.0%)	1 (3.3%)	11 (18.6)%	8 (13.1%)	51 (13.6%)	51 (16.7%)
45	0 (0.0%)	0 (0.0%)	0 (0.0%)	0 (0.0%)	2 (0.5%)	2 (0.7%)
47	0 (0.0%)	0 (0.0%)	0 (0.0%)	0 (0.0%)	0 (0.0%)	2 (0.7%)
48	0 (0.0%)	0 (0.0%)	2 (3.4%)	0 (0.0%)	6 (1.6%)	3 (1.0%)
49	1 (1.1%)	1 (3.3%)	0 (0.0)%	1 (1.6%)	7 (1.9%)	3 (1.0%)
50	2 (2.3%)	0 (0.0%)	2 (3.4%)	1 (1.6%)	9 (2.4%)	7 (2.3%)
51	18 (20.5%)	8 (26.7%)	16 (27.1%)	14 (23.0%)	59 (15.7%)	34 (11.1%)
52	15 (17.0%)	0 (0.0%)	6 (10.2%)	6 (9.8%)	37 (9.8%)	36 (11.8%)
53	0 (0.0%)	1 (3.3%)	1 (1.7%)	0 (0.0%)	9 (2.4%)	10 (3.3%)
55	2 (2.3%)	1 (3.3%)	1 (1.7%)	2 (3.3%)	6 (1.6%)	5 (1.6%)
56	3 (3.4%)	0 (0.0%)	0 (0.0)%	0 (0.0%)	7 (1.9%)	3 (1.0%)
57	19 (21.6%)	2 (6.7%)	1 (1.7%)	4 (6.6%)	48 (12.8%)	29 (9.5%)
58	4 (4.5%)	1 (3.3%)	3 (5.1%)	3 (4.9%)	33 (8.8%)	25 (8.2%)
40	23 (26.1%)	10 (33.3%)	8 (13.6%)	12 (19.7%)	13 (3.5%)	13 (4.2%)
60	6 (6.8%)	1 (3.3%)	3 (5.1%)	1 (1.6%)	26 (6.9%)	14 (4.6%)
61	17 (19.3%)	9 (30.0%)	5 (8.5%)	11 (18.0%)	51 (13.6%)	38 (12.4%)
62	8 (9.1%)	0 (0.0%)	4 (6.8%)	3 (4.9%)	18 (4.8%)	15 (4.9%)
63	0 (0.0%)	0 (0.0%)	1 (1.7%)	0 (0.0%)	2 (0.5%)	4 (1.3%)
65	0 (0.0%)	0 (0.0%)	0 (0.0)%	0 (0.0%)	1 (0.3%)	0 (0.0%)
70	0 (0.0%)	0 (0.0%)	0 (0.0)%	0 (0.0%)	12 (3.2%)	7 (2.3%)
71	1 (1.1%)	0 (0.0%)	1 (1.7%)	1 (1.6%)	4 (1.1%)	11 (3.6%)
75	2 (2.3%)	1 (3.3%)	1 (1.7%)	3 (4.9%)	8 (2.1%)	6 (2.0%)
77	0 (0.0%)	0 (0.0%)	0 (0.0%)	0 (0.0%)	1 (0.3%)	0 (0.0%)
78	0 (0.0%)	0 (0.0%)	0 (0.0%)	0 (0.0%)	1 (0.3%)	1 (0.3%)
81	0 (0.0%)	0 (0.0%)	0 (0.0%)	0 (0.0%)	3 (0.8%)	4 (1.3%)

**Table:** (c)

	HIV positive females		HIV positive males		Healthy renal donors	
HLA DR	TB neg. F	TB pos. F	TB neg. M	TB pos. M	Females	Males
	*n* = 88	*n* = 30	*n* = 59	*n* = 61	*n* = 376	*n* = 306

1	5 (5.7%)	3 (10.0%)	6 (10.2%)	5 (8.2%)	17 (4.52%)	19 (6.21%)
3	0 (0.0%)	0 (0.0%)	1 (1.7%)	0 (0.00%)	9 (2.39%)	9 (2.94%)
4	20 (22.7%)	5 (16.7%)	17 (28.8%)	13 (21.3%)	39 (10.37%)	40 (13.07%)
7	22 (25.0%)	5 (16.7%)	8 (13.6%)	11 (18.0%)	72 (19.15%)	44 (14.38%)
8	3 (3.4%)	0 (0.0%)	1 (1.7%)	7 (11.5%)	3 (0.80%)	8 (2.61%)
9	0 (0.0%)	0 (0.0%)	1 (1.7%)	1 (1.6%)	0 (0.00%)	5 (1.63%)
10	11 (12.5%)	3 (10.0%)	9 (15.3%)	11 (18.0%)	36 (9.57%)	24 (7.84%)
11	9 (10.2%)	3 (10.0%)	6 (10.2%)	9 (14.8%)	20 (5.32%)	25 (8.17%)
12	3 (3.4%)	1 (3.3%)	4 (6.8%)	2 (3.3%)	14 (3.72%)	17 (5.56%)
13	13 (14.8%)	6 (20.0%)	12 (20.3%)	10 (16.4%)	47 (12.50%)	22 (7.19%)
14	20 (22.7%)	4 (13.3%)	11 (18.6%)	10 (16.4%)	34 (9.04%)	23 (7.52%)
15	47 (53.4%)	17 (56.7%)	26 (44.1%)	26 (42.6%)	105 (27.93%)	98 (32.03%)
16	4 (4.5%)	2 (6.7%)	1 (1.7%)	0 (0.00%)	4 (1.06%)	5 (1.63%)
17	3 (3.4%)	1 (3.3%)	3 (5.1%)	7 (11.5%)	9 (2.39%)	10 (3.27%)
18	0 (0.0%)	1 (3.3%)	0 (0.0%)	0 (0.00%)	0 (0.00%)	3 (0.98%)

*Using Chi-square test, the significance of frequency distribution of various HLA alleles was compared between males and females of HIV subjects with and with out TB as well as in healthy renal donors. The only significant observation noted was lower occurrence of TB in HLAB57 positive HIV infected women (*P* = 0.0046).
